# Calbindin-D9k Ablation Disrupt Glucose/Pancreatic Insulin Homeostasis

**DOI:** 10.1371/journal.pone.0164527

**Published:** 2016-10-13

**Authors:** Changhwan Ahn, Dongoh Lee, Jae-Hwan Lee, Hyun Yang, Beum-Soo An, Eui-Bae Jeung

**Affiliations:** 1 Laboratory of Veterinary Biochemistry and Molecular Biology, Veterinary Medical Center and College of Veterinary Medicine, Chungbuk National University, Cheongju, Chungbuk 28644, Republic of Korea; 2 Department of Biomaterials Science, College of National Resources & Life Science, Pusan National University, Miryang, Gyeongsangnam-do 627-706, Republic of Korea; University of North Dakota School of Medicine and Health Sciences, UNITED STATES

## Abstract

It has been proposed that cellular Ca^2+^ signals activate hormone secretion. In pancreatic β cells, which produce insulin, Ca^2+^ signals have been known to contribute to insulin secretion. Prior to this study, we confirmed that insulin-secreting β cells express CaBP-9k, and assumed that CaBP-9k play a role in β cell insulin synthesis or secretion. Using CaBP-9k knock out (KO) mice, we demonstrated that ablation of CaBP-9k causes reducing insulin secretion and increasing serum glucose. To compare the role of CaBP-9k with pathophysiological conditions, we exposed wild-type and CaBP-9k KO mice to hypoxic conditions for 10 days. Hypoxia induced endoplasmic reticulum (ER) stress, increasing both insulin signaling and insulin resistance. By exposing hypoxia, CaBP-9k KO mice showed an increased level of ER stress marker protein relative to wild type mice. Without hypoxic conditions, CaBP-9K ablation regulates calcium channels and causes ER stress in a CaBP-9K specific manner. Ablation of CaBP-9k also showed decreased levels of sulfonylurea receptor1 (SUR1) and inward-rectifier potassium ion channel 6.2 (K_ir_6.2), which are insulin secretion marker genes. Overall, the results of the present study demonstrated that CaBP-9k regulates synthesis of insulin and is part of the insulin-secreting calcium signaling.

## Introduction

Calbindin-D_9k_ (CaBP-9k) is a 9-kDa polypeptide with two EF-calcium-binding sites that are usually expressed in the mammalian intestine, uterus, and pituitary gland. The major role of this protein is buffering of Ca^2+^ ions [1].

The discovery of a high affinity receptor in the pancreas for the hormonally active form of vitamin D, 1,25-dihydroxyvitamin D3 (1,25(OH)_2_D_3_), in 1979 was the first demonstration of a nonclassical target tissue to contain vitamin D receptors (VDR) [2]. Impaired insulin secretory capacity in pancreatic β cells was also observed in mice lacking a functional VDR [3]. 1,25(OH)_2_D_3_ and its cognitive VDR regulate many target genes, including CaBP-9k. The expression of CaBP-9k has been shown to be mediated by vitamin D response element (VDRE) on its promoter. CaBP-9k regulates the amount of intracellular calcium to prevent cell death from reaching the toxicity of free calcium [4].

Calcium signaling in all secretory cells (nerve cell, endocrine and exocrine cells) is mediated by exocytosis requiring ATP, Ca^2+^ and Mg^2+^ [5]. In insulin-secreting β cells, Ca^2+^channels cause Ca^2+^ influx and induce increases in cytosolic Ca^2+^ concentrations, which activates exocytotic insulin secretion [6]. Increases in matrix Ca^2+^ induces amplification of sustained glucose-dependent insulin secretion in β cells [7]. Another mechanism regulating intracellular Ca^2+^ concentration is the endoplasmic reticulum (ER) mediated pathway by which Ca^2+^ moves across the ER membrane via calcium channels [8]. Cellular free Ca^2+^ and ER Ca^2+^ are modulated by several calcium channels including the sarcoplasmic reticulum Ca^2+^ ATPase (SERCA)2a and 2b, inositol 1,4,5-trisphosphate receptor (IP_3_R), and ryanodine receptor 2 (RyR2). The results showed that Ca^2+^ is taken up into the ER by an electrogenic Ca^2+^ pump (i.e., SERCA2a and 2b), whereas RyR2 and IP_3_ acts on the ER membrane by opening up a Ca^2+^-permeable conductance to cytoplasm [8, 9]. Disruption of intracellular Ca^2+^ homeostasis can trigger ER stress. Recently, overexpression of the calcium efflux channel, plasma membrane Ca^2+^-ATPase (PMCA), was found to deplete ER calcium storage, leading to ER stress and apoptosis [10]. ER stress is a cellular response related to the ER. Following ER stress, the ER attempts to restore normal function by halting protein translation, degrading misfolded proteins, and increasing production of chaperones involved in protein folding [9, 11].

Insulin-resistant states such as Type-2 Diabetes (T2D) causes a burden to the pancreas, especially on insulin-secreting β cells, owing to the synthesis and secretion of higher amounts of insulin. This burden creates ER stress, which triggers suppression of insulin receptor signaling [12]. Diabetes is a complex disease characterized by both insulin resistance and β-cell dysfunction [13]. In this study, we generated an animal model by inducing hyperglycemia through hypoxic stress. Under this pathological condition, we evaluated the function of CaBP-9k in the pancreas using CaBP-9k knock out (KO) mice. In addition, we examined the relationship of CaBP-9k with glucose level, insulin resistance and β cell function in the presence or absence of hypoxic stress.

## Materials and Methods

### Animal experiments

Male C57BL6 mice, weighing 25~30 g, nine weeks of age, were obtained from Samtako (Osan, Gyeonggi, Republic of Korea). All animals were housed in polycarbonate cages and acclimated in an environmentally controlled room (temperature: 23±2°C, relative humidity: 50±10%, frequent ventilation, and a 12-h light/dark cycle). After approximately one week of acclimatization, the mice were divided into four groups; Wild-type mice with normoxic condition (group 1), Wild-type mice with hypoxic condition (group 2), CaBP-9k KO mice with normoxic condition (group 3) and CaBP-9k KO mice with hypoxic condition (group 4). Rooms for giving hypoxic condition polyacryl cages with controlled oxygen concentrations (20% ± 2% for normoxia and 12% ± 2% for hypoxia) were used. Mice in hypoxia group were maintained with a constant inspired fraction of 10% oxygen for 10 days. After 10 days, all the mice were anesthetized by Inhalation of isoflurane for collecting blood sample. After collecting blood, mice were euthanized by cervical disclosure. Institutional Animal Care and Use Committee (IACUC) of Chungbuk National University approved all experimental procedures.

### Immunofluorescence assay

The 4μm slides of formalin fixed paraffin embedded pancreas tissue were deparaffinized with xylene and rehydrated with ethanol. Antigen retrieval was perfumed with 0.05% citrate buffer, samples were block in 5% goat serum and incubated with CaBP-9k (Swant, Bellinzona, Switzland; 1:1000) and insulin (Santa Cruz Biotech. Co., CA, USA; 1:1000) antibody. After washing, samples were incubated with Dylight 550 goat-anti-rabbit IgG (1:500, (Thermo Fisher Scientific, MA, USA). Nuclei were stained with 40,6-diamidino-2-phenylindole (DAPI, Roche, Basel, Switzerland).

### Collection and serological analysis of serum

Blood was collected from each mice, transferred to serum separator tubes (Microtainer tubes; Becton-Dickinson Co., NJ, USA), centrifuged at 400×g for 15 min, and aliquoted as 200μl. Serum glucose are analyzed using the glucometer AccuChek^®^ Active (Roche Diagnostics GmbH, Mannheim, Germany). The animals were fasted for 4 hours before performance of blood glucose measurements. Plasma insulin level was also determined, by using the insulin ELISA kit (SHIBAYAGI, Japan) according to the manufacturer’s instructions.

### Calculation of HOMA-IR index

The homeostatic model assessment (HOMA) is a method used to quantify insulin resistance and β cell function. The calculation of HOMA-IR were used formula “HOMA-IR = (Glucose level (mg/dl) * Insulin level (ng/ml)/405”

### Glucose/insulin tolerance test

*Intraperitoneal Glucose Tolerance Test (IPGTT)*: Prior to glucose administration, mice underwent a 6 hour fast to achieve a baseline blood glucose level. After the fasting time a 2 mm distal section of the mouse’s sterilized tail is snipped using a scalpel and gently squeezed to obtain a drop of blood. The blood drop is applied directly to an AccuChek^®^ Active (Roche Diagnostics GmbH, Mannheim, Germany) to obtain a baseline T_0_ blood glucose reading expressed as mg/dL. Then after 20% glucose solution was injected (2g of glucose/kg body mass) intraperitoneally, blood glucose level at 30, 60 and 120min were measured.

*Intraperitoneal Insulin Tolerance Test (IPITT)*: Prior to Insulin administration, fast mice for 4 hours early in the morning (7:00 a.m.). Using same methods of IPGTT, a drop of blood is applied for baseline T_0_ blood glucose reading expressed as mg/dL. Give the mice an intraperitoneal injection of insulin (0.5U/kg). Continue to take blood samples from T_0,_ blood glucose at 30, 60 and 120 min.

### Total RNA extraction and quantitative real-time PCR

Mice were euthanized, and the pancreas tissues rapidly exercised and washed in cold, sterile NaCl (0.9%). Total RNA was prepared with TRIzol reagent (Invitrogen, Carlsbad, CA), and the concentration of RNA was determined by absorbance at 260 nm. Total RNA (1 μg)was reverse transcribed into first-strand cDNAs using Moloney murine leukemia virus (MMLV) reverse-transcriptase (iNtRON Bio, Sungnam, Gyeonggi, Korea) and random primers (9-mers; TaKaRa Bio. Inc., Otsu, Shiga, Japan). 2 μl of cDNA template was added to 10 μl of 2SYBR Premix Ex Taq (TaKaRa Bio) and 10 pmol of each specific primer. The reactions were carried out for 40 cycles according to the following parameters: denaturation at 95°C5for 30 sec, annealing at 58°Cofor 30 sec, and extension at 72°Cofor 30 sec. Fluorescence intensity was measured at the end of the extension phase of each cycle. The threshold value for the fluorescence intensity of all samples was set manually. The reaction cycle at which PCR products exceeded this fluorescence intensity threshold was identified as the threshold cycle in the exponential phase of the PCR amplification. The expressions of target genes were quantified against that of β-actin. Relative quantification was based on the comparison of CT at a constant fluorescent intensity. The amount of transcript is inversely related to the observed CT, and for every twofold dilution in the transcript, CT is expected to increase by 1. Relative expression was calculated using the equation R = 2^-(ΔCTsample -ΔCTcontrol^. To determine a normalized arbitrary value for target gene expression, its expression level was normalized to that of β-actin.

### Western blot analysis

The pancreas of euthanized mice were rapidly excised and washed in cold sterile 0.9% NaCl solution. Protein was extracted with Pro-prep (InTron., Inc., Seoul, Korea) according to the manufacturer’s instructions. Protein (50 mg per lane) was separated on a 10% SDS–polyacrylamide gel electrophoresis (PAGE) and transferred to a polyvinylidene fluoride transfer membrane (Perkin Elmer Co., Wellesley, MA) in a TransBlot Cell (TE-22, Hoefer Co., CA, USA) according to the manufacturer’s protocol. The resulting blot was blocked in TBS-T containing 5% skim milk for 60 min, then incubated in target primary antibody or β-actin (rabbit-monoclonal, 1:2,000, Assay Design, Inc., CA, USA) for 4 hr at room temperature. After washing in buffer, the membranes were incubated with the appropriate horseradish peroxidase-conjugated secondary antibodies (anti-rabbit, 1:2,000, SantaCruz, or anti-mouse, 1:5,000, Santa Cruz) for 1 hr at room temperature (RT). After washing, the blots were developed by incubation in ECL chemiluminescence reagent (Santa Cruz) and subsequently exposed to Biomax Light film (Kodak) for 1–5 min. Signal specificity was confirmed by blotting in the absence of primary antibody, and bands were normalized to β-actin immunoreactive bands visualized in the same membrane after stripping. Density measurements for each band were performed with NIH Image J software. Background samples from an area near each lane were subtracted from each band to obtain mean band density.

### Statistical analysis

The results of all experiments are presented as the mean ± SD. The number of mice for each group was 8. Data were analyzed with a nonparametric one-way analysis of variance (ANOVA), using the Tukey’s test for multiple comparisons and non-parametric two-way ANOVA. Data were ranked according to these tests. For power and sample size calculation, variation of value for a given settings of false positive rates (α) and power (1-β). All statistical analyses were performed using Graphpad^™^ software. P<0.05 was considered statistically significant.

## Results

### Comparison of glucose parameters under hypoxic condition after ablation of CaBP-9k

**Fig 1:**
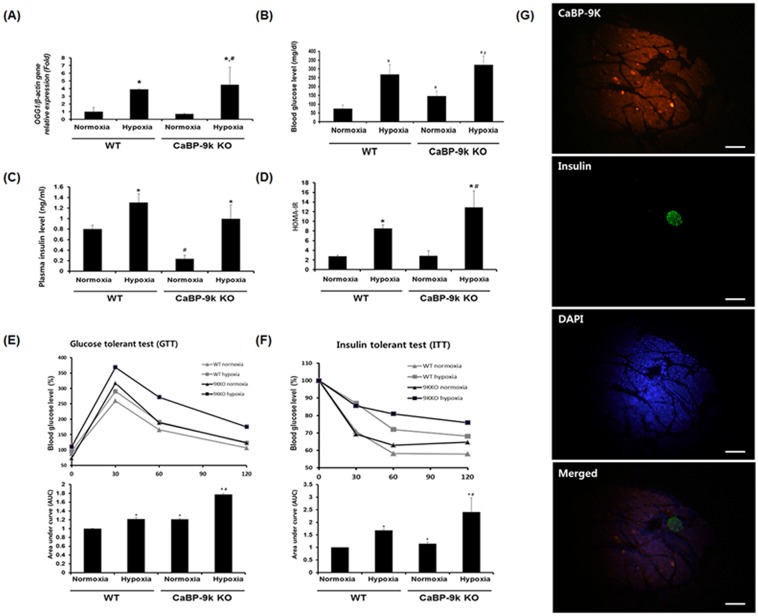
Regulation of glucose parameters in hypoxia induced hyperglycmic model and CaBP-9K KO mice. The mice were divided into four groups (n = 8 per each group, I: normoxia-wildtype, II: hypoxia-wildtype, III: normoxia-CaBP-9k KO, IV: hypoxia-CaBP-9k KO). (A) OGG1 expression for confirmation of hypoxia induction. (B) Measurement of blood glucose level. (C) Measurement of plasma insulin level. (D) Calculated insulin resistance index; HOMA-IR. (E) IPGTT and AUC. AUC was calculated by trapezoidal method with graphpad prism. (F) IPITT and AUC. (G) Immunofluorescence assay with CaBP-9k, insulin and DAPI. The pancreas was immunostained with insulin and CaBP-9K antibodies. The tissue sections were also stained with DAPI for counter staining. * indicates *P*<0.05 compared with normal oxygen conditioned group; ^#^ indicates *P*<0.05 compared between Wildtype-CaBP-9k KO.

To induce the hyperglycemic animal model, we exposed WT and CaBP-9k KO mice to hypoxic conditions by housing them in a hypoxia chamber for 10 days. To confirm hypoxic conditions, we examined expression of the hypoxia marker gene, 8-Oxoguanine glycosylase 1 (OGG1). As expected, OGG1 mRNA level was increased under hypoxia (Fig 1A).

Glucose parameters such as serum glucose and insulin levels were also measured to determine whether hypoxic stress successfully induces hyperglycemic conditions. Interestingly, serum glucose and insulin levels were significantly altered in CaBP-9k KO mice relative to WT, although hypoxic stress was not present. In CaBP-9k KO mice, serum glucose was increased, while insulin levels were significantly decreased compared to WT mice (Fig 1B and 1C). We next indirectly examined insulin resistance by measuring HOMA-IR (Fig 1D). The HOMA-IR value was enhanced under hypoxic conditions, which was more obvious in CaBP-9k KO mice.

### Glucose and insulin tolerance test under hypoxic conditions after CaBP-9k ablation

We conducted intraperitoneal glucose tolerance tests (IPGTT) in a hyperglycemic model with hypoxic condition after CaBP-9k ablation (Fig 1E). In the glucose tolerance test, hypoxia led to moderate changes in the in glucose levels in WT and CaBP-9k ablation mice, while drastically impaired glucose tolerance was observed in CaBP-9k KO mice.

Similar to the results of IPGTT, the intraperitoneal insulin tolerance test (IPITT) revealed impaired insulin tolerance in WT mice exposed to hypoxia, and this pattern was stronger in CaBP-9k KO mice (Fig 1F).

### CaBP-9k protein was expressed in insulin-secreting pancreatic β cells

To determine whether CaBP-9k is expressed in insulin-secreting β cells, we conducted dual immunofluorescence staining. Specifically, we stained CaBP-9k in combination with insulin and found that CaBP-9k co-localized with insulin, suggesting that insulin-secreting β cells also express CaBP-9k protein (Fig 1G).

### Regulation of insulin-related transcription factors under hyperglycemia with hypoxic condition after CaBP-9k ablation

**Fig 2:**
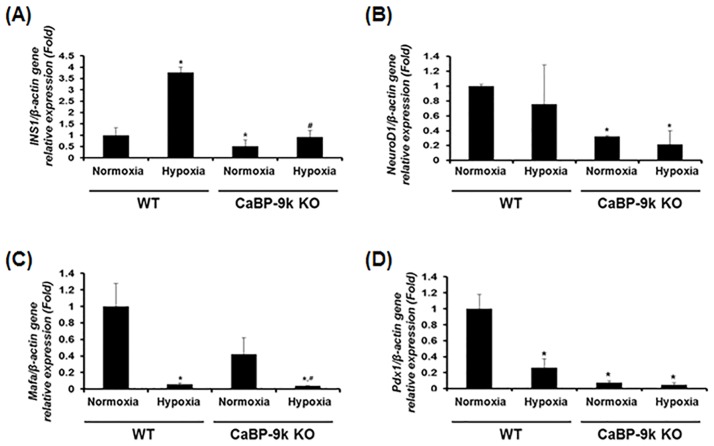
Expression of insulin-associated transcription factors in hypoxia induced hyperglycemic model and CaBP-9K KO mice. WT and CaBP-9K KO mice were exposed to normoxic or hypoxic condition for 10 days (n = 8 per each group). The mRNA was extracted from pancreas tissues and the transcriptional levels of INS1 (A), NEUROD (B), MafA (C), and PDX-1 (D) genes were analyzed by real-time PCR. Results were normalized relative to β-actin. * indicates *P*<0.05 compared with normal oxygen conditioned group; ^#^ indicates *P*<0.05 compared between Wildtype-CaBP-9k KO.

Since the amount of insulin in the serum was regulated by hypoxia and CaBP-9k, we verified mRNA levels of transcription factors regulating the expression of insulin gene in the pancreas. Transcriptional regulators of insulin gene expression include insulin 1 (INS1), neuronal differentiation 1 (NeuroD1), v-maf avian musculoaponeurotic fibrosarcoma oncogene homolog A (Mafa), and pancreatic and duodenal homeobox 1 (Pdx-1) [11]. Under hypoxic condition, the expression of INS1 was stimulated, whereas that of NeuroD1, Mafa, and Pdx1 was down regulated. In CaBP-9k KO mice, all tested transcription factors were down regulated relative to WT mice. When hypoxic stress was applied to CaBP-9k KO mice, INS1 was enhanced, whereas Mafa was reduced relative to normoxia. The expression of NeuroD1 and Pdx1 in CaBP-9k KO mice was not significantly changed by hypoxic conditions. (Fig 2)

### Expression of calcium modulating genes under hypoxic condition after CaBP-9k ablation

**Fig 3:**
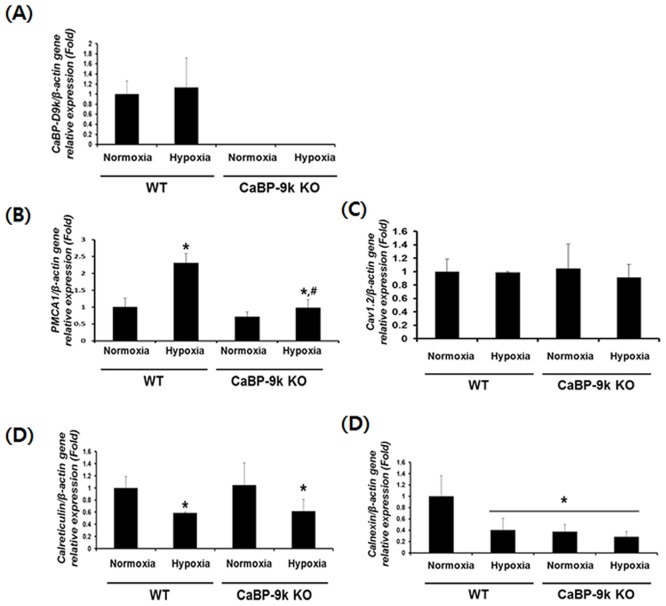
Expression of calcium modulating genes in hypoxia induced hyperglycemic model and CaBP-9K KO mice. WT and CaBP-9K KO mice were exposed to normoxic or hypoxic condition for 10 days (n = 8 per each group). The mRNA was extracted from pancreas tissues and the transcriptional levels of CaBP-9k (A), PMCA1 (B), Cav1.2 (C), CALR (D) and CANX (E) genes were analyzed by real-time PCR. Results were normalized relative to β-actin. * indicates *P*<0.05 compared with normal oxygen conditioned group; ^#^ indicates *P*<0.05 compared between Wildtype-CaBP-9k KO.

To examine the expression patterns of cytosolic calcium ion modulating molecules, we conducted real-time PCR of samples from the pancreas of hypoxia induced hyperglycemic mice. The calcium buffering gene CaBP-9k (Fig 3A), plasma membrane Ca2+-ATPase 1 (PMCA1, Fig 3B), voltage gated calcium influx channel C_av_1.2 (C_av_1.2, Fig 3C) and two chaperone genes, calreticulin (CALN, Fig 3D) and calnexin (CANX, Fig 3E), were evaluated. CaBP-9k was not detected in KO mice and the transcripts were not altered under hypoxic conditions. Calcium efflux channel, PMCA1, was increased under hypoxic conditions, whereas, calcium influx channel, Cav1.2, was not altered by hypoxia. In CaBP-9k KO mice, Cav1.2 was also not changed, while the effects of hypoxia on the increase of PMCA1 was significantly reduced compared with WT mice.

Since PMCA1 is known to be an ER-stress marker, the chaperone and ER quality control genes CALN and CANX were also tested. Both CALN and CANX decreased under hypoxia. Interestingly, under normoxic conditions, CANX transcripts showed similar patterns as observed under hypoxia in CaBP-9k KO mice, suggesting that CaBP-9k KO and hypoxia induced hyperglycemic condition gave rise to ER-stress.

### Expression of ER-stress maker genes under hypoxic conditions after CaBP-9k ablation

**Fig 4:**
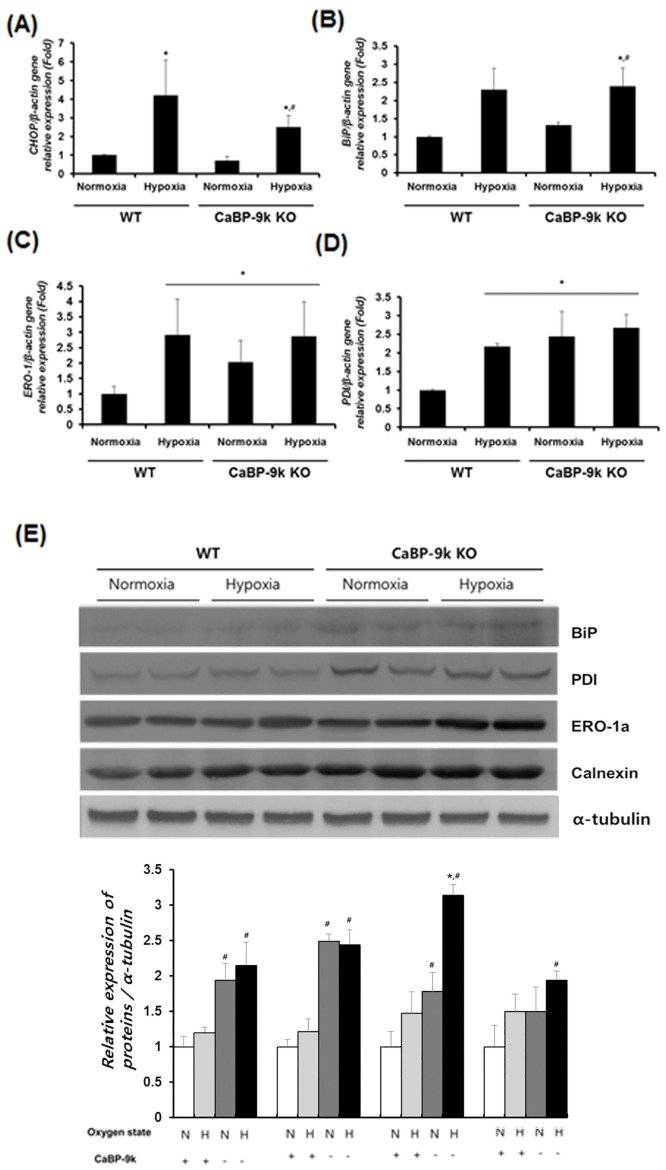
Expression of ER-stress maker genes in hypoxia induced hyperglycemic model and CaBP-9K KO mice. WT and CaBP-9K KO mice were exposed to normoxic or hypoxic condition for 10 days (n = 8 per each group). The mRNA was extracted from pancreas tissues and the transcriptional levels of CHOP (A), BiP (B), ERO1 (C), and PDI (D) genes were analyzed by real-time PCR. (E) Western blot analysis of BiP, PDI, ERO-1a and Calnexin was performed for evaluating protein levels of ER stress marker genes. Results were normalized relative to β-actin. * indicates *P*<0.05 compared with normal oxygen conditioned group; ^#^ indicates *P*<0.05 compared between Wildtype-CaBP-9k KO.

The transcriptional expression of genes involved in ER stress were examined under hypoxic conditions after CaBP-9k ablation (Fig 4). Real-time PCR revealed that C/EBP-homologous protein (CHOP), immunoglobulin-heavy-chain-binding protein (BiP), ER oxidoreductin 1 (ERO1) and protein disulfide isomerase (PDI) genes responsible for ER stress were elevated under hypoxic conditions. In CaBP-9k KO mice, ERO-1 and PDI were enhanced even though hypoxic stress was not applied. Western blot analysis was conducted to confirm the transcriptional regulation of ER stress genes. The results of western blot analysis revealed the same tendency as those of mRNA, with increasing CHOP, BiP, ERO-1, and PDI protein levels and decreasing calnexin under hypoxic conditions and CaBP-9k ablation. These results demonstrate that hypoxic conditions and CaBP-9k ablation induce ER-stress in the pancreas.

### Expression of ER-associated calcium modulating genes under hypoxic conditions after CaBP-9k ablation

**Fig 5:**
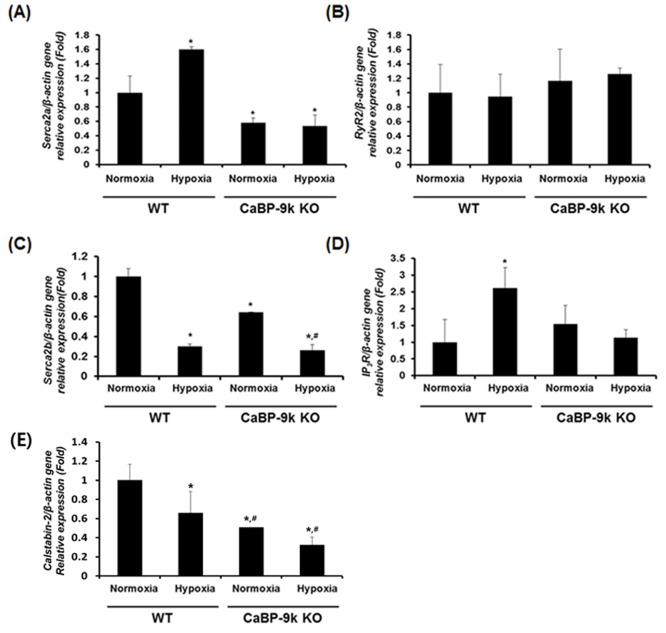
Expression of ER-calcium modulating genes in hypoxia induced hyperglycemic model and CaBP-9K KO mice. WT and CaBP-9K KO mice were exposed to normoxic or hypoxic condition for 10 days (n = 8 per each group). The mRNA was extracted from pancreas tissues and the transcriptional levels of SERCA2a (A), SERCA2b (B), RyR2 (C), IP3R (D), SUR1 (E), and K_ir_6.2 (F) genes were analyzed by real-time PCR. Results were normalized relative to β-actin. * indicates *P*<0.05 compared with normal oxygen conditioned group; ^#^ indicates *P*<0.05 compared between Wildtype-CaBP-9k KO).

Calcium homeostasis mediated by ER is crucial for normal cell function, including synthesis and secretion of endogenous molecules and muscle contractions. Since PMCA1 calcium efflux channel and ER stress were regulated in our results, we further examined ER-associated calcium regulating genes in CaBP-9k KO and WT mice in the absence and presence of hypoxic conditions. The sarcoplasmic/endoplasmic reticulum Ca^2+^ pump (SERCA), which is responsible for calcium influx to the ER and ryanodine receptor 2 (RyR2)/Inositol 1,4,5-Trisphosphate Receptor (IP3R) responsible for calcium efflux from the ER were examined (Fig 5). SERCA2b, the major isoform in the pancreas, but not SERCA2a was down-regulated under hypoxic conditions and CaBP-9k ablation. In contrast, up-regulation of IP_3_R under hypoxic conditions was observed. These results demonstrated that calcium in the ER was depleted under hypoxic conditions and by ablation of CaBP-9k, which caused ER-stress in the pancreas.

### Expression of insulin secretion K_ATP_ channel under hypoxic conditions after CaBP-9k ablation

**Fig 6:**
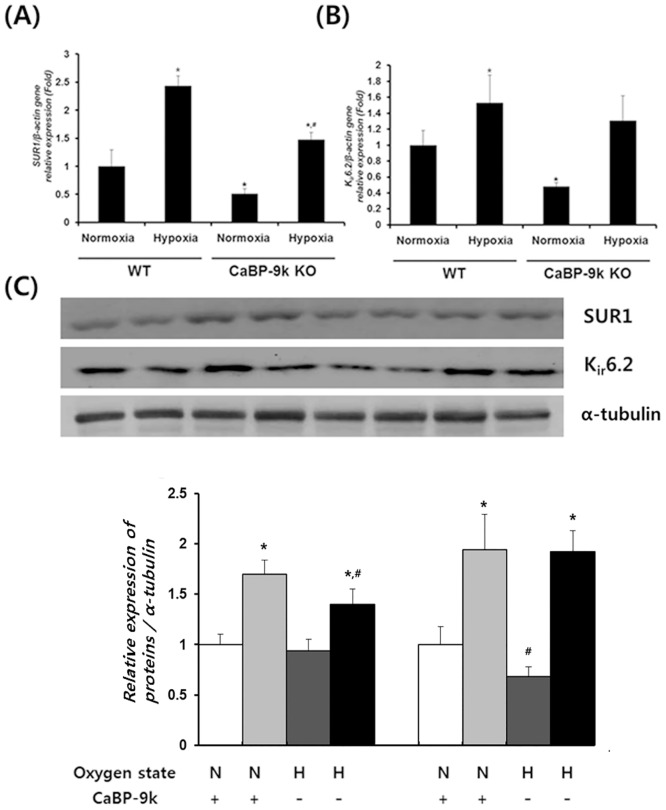
Expression of insulin secretion related K_ATP_ channel in pancreas. (A)–Sulfonylurea receptor1 (SUR1) (B)–ATP-sensitive K^+^ channel, an inward-rectifier potassium ion channel 6.2 (K_ir_6.2) mRNA expression were examined with realtime PCR. Results were normalized relative to β-actin. (* indicates *P*<0.05 compared with normal oxygen conditioned group, ^#^ indicates *P*<0.05 compared between Wildtype-CaBP-9k KO).

In pancreatic β-cells, glucose stimulates the entry of extrinsic Ca^2+^ through closure of the K_ATP_ channels and depolarization of the β cell membrane, leading to an increase of intracellular Ca^2+^ and finally induction of insulin exocytosis. Therefore, regulation of K_ATP_ subunits was evaluated (Fig 6). Subunits of the K_ATP_ channel, sulfonylurea receptors (SUR1) and K*ir* 6.2, were decreased in the pancreas of CaBP-9k KO mice, while hypoxic stress itself increased the mRNA levels of the genes. These findings were consistent with the results observed for serum insulin (Fig 1C).

## Discussion

The correlation between vitamin D and pancreatic function has been relatively well-studied. A recent study demonstrated that vitamin D-deficient rats showed increased β-cell sensitivity to 1,25(OH)2D3-dependent insulin secretion under hypocalcemic conditions [14]. CaBP-9k is known to be a vitamin-D-dependent calcium-binding protein; however, the role of CaBP-9k in inulin synthesis and secretion by the pancreas has not been explored. In the present study, we investigated the role of CaBP-9k under normal and hypoxic conditions. For this study, hypoxic pathological conditions were defined by the glucose parameter and glucose/insulin tolerance test. To verify the role of CaBP-9k in insulin-secreting β-cells, we performed hematological examinations by measuring the serum glucose level and plasma insulin level. In the absence of CaBP-9k protein, serum glucose levels increased, which was similar to the results observed under hypoxia-induced pathologic conditions. Interestingly, insulin was differently regulated by CaBP-9k and hypoxia by decreased in CaBP-9k KO mice, while it was increased by hypoxic conditions. These results suggest that the pathophysiological conditions of the CaBP-9k KO model differ from those of T2D. Plasma insulin was reduced by CaBP-9k ablation, suggesting that CaBP-9k KO mice are closer to the model of Type 1 diabetes than T2D. This hypothesis was further evidenced by GTT and ITT data, which showed that blood glucose levels were synergistically elevated by hypoxic conditions and CaBP-9k ablation. Furthermore, CaBP-9k was found to be colocalized with insulin upon immunostaining. These results suggest that CaBP-9k ablation elevates serum glucose through a different mechanism of T2D, which may be a type 1 diabetes-related mechanism. To examine whether these phenomena were caused by decreased insulin production or insulin resistance, we calculated the HOMA-IR values, which confirmed that glucose regulation was derived from insulin production rather than insulin resistance. In addition, insulin regulators such as MafA, NEUROD1, PDX-1 and insulin transcript INS1 showed the same tendencies. Because insulin production is regulated by calcium-related exocytosis [15], we tested calcium transporting proteins. Unlike calcium influx channel C_av_1.2, calcium efflux PMCA1 channel was up-regulated by hypoxia and down-regulated by CaBP-9k ablation, consistent with results of serum insulin. A previous study showed that overexpressed PMCA induces ER stress and apoptosis [10]. Because ER is related to intracellular calcium concentration in pancreatic β-cells, we monitored ER conditions by detecting the expression of CALR and CANX, which are known ER quality control genes [16, 17]. Hypoxic conditions reduced ER quality via decreased CALR expression. Moreover, CANX mRNA levels were decreased by CaBP-9k ablation, suggesting that CaBP-9K is involved in the control of ER quality.

Glucose intolerant or insulin-resistant states such as early T2D cause a burden on the pancreas, especially insulin-secreting β cells, owing to the need to synthesize and secrete more insulin to maintain normal glucose tolerance [18, 19]. This burden creates ER stress, which triggers insulin resistance. Thus, disrupted or hampered calcium metabolism in calcium signaling could result in ER stress and insulin resistance or decreased insulin secretion [20–22].

We next verified expression of ER stress-related marker genes. Hypoxic hyperglycemic conditions induced ER stress by regulating CHOP, BiP, ERO-1 and PDX. CaBP-9k ablation itself caused ER stress, regardless of hypoxic condition, by stimulating expression of ERO-1 and PDX. This may have exerted a negative effect on insulin production due to inability to intreacellular calcium buffering function. To maintain intracellular calcium homeostasis, ER stress are results in CaBP-9k ablation. It is well known that ER stress is associated with calcium influx and efflux channels. In the cardiomyocyte, CANX silencing affects SERCA and RyR2, which are responsible for the lumen of the ER and calcium concentration [9]. SERCA 2a and 2b, which are responsible for calcium influx from the cytosol to ER [12], were examined in this study. During regulation of Serca2a, the results were similar to those observed for serum insulin. mRNA expression of Serca2a was increased by hypoxia, while it decreased following CaBP-9k ablation, again suggesting that CaBP-9K is related to T1D like conditions. CaBP-9k ablation also resulted in decreased SERCA2b relative to WT mice. IP_3_R expression was only increased in hypoxia in wild-type mice. Decreased calcium influx channel in the ER membrane represents reduced calcium concentration in the ER, which causes ER-stress condition [9, 23]. It has been reported that impaired ER protein trafficking by ER stress leads to impaired pro-insulin maturation and loss of insulin content [24, 25].

To examine the relationship between CaBP-9K and insulin secretion, we examined expression of SUR1 and K*ir* 6.2, which are involved in the insulin secretion pathway [26]. Both SUR1 and K*ir* 6.2 showed decreased levels after CaBP-9k ablation, while they were stimulated by hypoxic conditions.

In summary, we identified the relevance of CaBP-9k to insulin production under normal and hypoxic conditions. CaBP-9k ablation revealed a different physiological condition from the T2D model. CaBP-9K ablation inhibited synthesis of insulin by reducing its transcription factors, including INS1, NeuroD1, Mafa, and Pdx1. The secretion of insulin was impaired by calcium concentration derived from calcium channel proteins including CANX and Serca2a, as well as ER stress. CaBP-9K-specific ER stress was caused by ERO-1a and PDI gene regulation. CaBP-9K-specific insulin secretion was also associated with the K_ATP_ channel subunits, SUR1 and K*ir* 6.2. Finally, CaBP-9K ablation caused T1D pathological conditions by reducing insulin production and induction of serum glucose. These results indicate that CaBP-9K is closely associated with the production and secretion of insulin in pancreas cells. Therefore, dysregulation of CaBP-9K signaling may be associated with diabetes mellitus.
